# The international partner universities of East African health professional programmes: why do they do it and what do they value?

**DOI:** 10.1186/s12992-019-0477-7

**Published:** 2019-06-07

**Authors:** Aaron N. Yarmoshuk, Donald C. Cole, Anastasia Nkatha Guantai, Mughwira Mwangu, Christina Zarowsky

**Affiliations:** 1 School of Public Health, University of the Western Cape, Cape Town, South Africa; 2 Dalla Lana School of Public Health, University of Toronto, Toronto, Canada; 3 University of Nairobi, Nairobi, Kenya; 4 Muhimbili University of Health and Allied Sciences, Dar es Salaam, Tanzania; 5 CR-CHUM/ESPUM, Université de Montréal, Montréal, Canada

**Keywords:** Partnerships, Global Health, Education, Research, Service, High education, University rankings, Social responsibility, Internationalization, Globalization

## Abstract

**Background:**

Globalization and funding imperatives drive many universities to internationalize through global health programmes. University-based global health researchers, advocates and programmes often stress the importance of addressing health inequity through partnerships. However, empirical exploration of perspectives on why universities engage in these partnerships and the benefits of them is limited.

**Objective:**

To analyse who in international partner universities initiated the partnerships with four East African universities, why the partnerships were initiated, and what the international partners value about the partnerships.

**Methods:**

Fifty-nine key informants from 26 international universities partnering with four East African universities in medicine, nursing and/or public health participated in individual in-depth interviews. Transcripts were analysed thematically. We then applied Burton Clark’s framework of “entrepreneurial” universities characterized by an “academic heartland”, “expanded development periphery”, “managerial core” and “expanded funding base”, developed to examine how European universities respond to the forces of globalization, to interpret the data through a global health lens.

**Results:**

Partnerships that were of interest to universities’ “academic heartland” - research and education - were of greatest interest to many international partners, especially research intensive universities. Some universities established and placed coordination of their global health activities within units consistent with an expanded development periphery. These units were sometimes useful for helping to establish and support global health partnerships. Success in developing and sustaining the global health partnerships required some degree of support from a strengthened steering or managerial core. Diversified funding in the form of third-stream funding, was found to be essential to sustain partnerships. Social responsibility was also identified as a key ethos required to unite the multiple elements in some universities and sustain global health partnerships.

**Conclusion:**

Universities are complex entities. Various elements determine why a specific university entered a specific international partnership and what benefits it accrues. Ultimately, integration of the various elements is required to grow and sustain partnerships potentially through embracing social responsibility as a common value.

## Introduction/background

Globalization is one driver for the development of internationalization policies and practices among universities in the Global North and in the Global South and the development of partnerships among them. During the first fifteen years of this century, global health programmes in high-income countries (HICs), especially in United States universities, have grown rapidly [1–3]. While addressing the health inequalities between HICs and low- and middle-income countries (LMICs) appears to be a key motivation among many global health programmes, Macfarlane et al. [1] caution that “… the new academic programs in global health must be set within the growing trend towards the ‘internationalization of higher education’”.

In two previous papers, we mapped 125 distinct international university partnerships considered significant for increasing the capacity of health professional programmes (HPPs) of four universities in East Africa [4] and identified which of the partnerships were considered higher-value by these universities’ senior representatives [5] and why. In this paper we shift our attention to the international partners and examine responses to two questions. One, why did the international partners enter into the partnerships? Two, what do they perceive to be the benefits of the partnerships? First, however, we will introduce an analytical framework which we have found helpful for analysing partnerships.

### A framework for understanding who or what parts of a university are involved in internationalization?

Universities are complex organisations not simply because they are composed of multiple faculties or schools, that usually contain multiple departments themselves, but because the principal professionals working in them are professors who strongly desire “autonomy and freedom” [6]. Administration units and centres are also within universities. The importance of the “human factor” and “paying attention to individuals” in international university health partnerships was documented over twenty years ago [7]. Considering these individuals within a framework for examining universities may be helpful.

Examining the development of entrepreneurial universities in recent decades, sociologist of higher education Burton Clark argues that most universities need to diversify funding as core government support has been reduced at the same time as demand for higher education has increased as massification – “mass demand for higher education” [8] - has become an explicit goal in most countries even while knowledge obtainment has become more expensive [9–11]. Clark focuses on four elements of an “entrepreneurial university” [11]. The first is “a strengthened steering core”. Clark refers to the “steering core” as the “managerial core”, stating that it “ … consists of agents who work to find resources for the institution as a whole.” This includes a university’s central administration, deans and chairs. The second element is “a stimulated academic heartland”, the academic departments whose representatives lead and conduct education and research. Three, “an expanded development periphery” includes centres and outreach offices engaging stakeholders locally, nationally, regionally and globally. Fourth, “a diversified funding base” characterizes entrepreneurial universities, which seek and obtain funding from a variety of sources in addition to core funding from central government, whether federal or state/provincial. Clark refers to this as “third-stream” funding, or discretionary funding. It includes funding from non-traditional sources such as industrial firms, local governments, philanthropic foundations, royalty income from intellectual property, earned income from campus services, student fees, and alumni fundraising [11]. Because these four elements tend to diverge in priorities and modes of operating, their coordination (or lack thereof) is also important. Clark’s fifth element for examining the effectiveness of a university becoming entrepreneurial is the *integration*^1^ of the first four elements. If a university fails to integrate the four elements sufficiently well, it will not maximize its ability to become an entrepreneurial university. In the case of international health partnerships failure to integrate the four elements may result in a university’s global health work not reaching its potential. Using Clark’s first four elements may be useful when analysing who within universities establish partnerships with universities in East Africa and why, in the name of global health.

## Methodology

This was a qualitative study based on key informant interviews (KIIs) and subsequent qualitative analysis. It was part of a larger study which analysed 125 international partnerships identified by four East African universities as significant for strengthening their academic health programmes in medicine, nursing, and/or public health. We have previously reported on the perspectives of the four East African universities - Moi University (MU), University of Nairobi (UoN), Kilimanjaro Christian Medical University College (KCMUCo) and Muhimbili University of Health and Allied Sciences (MUHAS) [4]. This paper reports on the perspectives of the international partners.

### Who are the International Partners of the Four Focus Universities?

The general characteristics of the 125 partnerships identified by the four focus universities were reported previously [4]. In summary: i) over 70% of international partners came from high-income countries in Europe or North America; ii) all the African international partners were from neighbouring countries or middle-income^2^ countries; iii) seventy-two (72%) percent of the partnerships were of 10 years’ duration or less; iv) almost all, 92%, of the partnerships included education activities, approximately half, 47%, included research, and approximately a quarter, 24%, included service [4], Senior representatives of the focus universities perceived 25% of the partnerships they identified to be “higher-value” for strengthening one or more of their health professional programmes (HPPs) in some way [5].

### Participants

For the study reported here, fifty-nine (59) representatives from 26 universities on three continents (African *n* = 3, European *n* = 9, North American *n* = 14) were identified as key informants (KIs) and individually interviewed. These 59 KIs represented 30 of the 125 (24%) distinct partnerships, including some of the 10 consortia identified, of the four focus East African universities [4].

Representatives were selected based on a variety of criteria beginning with representatives from higher-value partnerships, as perceived by the senior representatives of the four focus universities. Approximately three-quarters, 23 of 31 (74%), of the higher-value partnerships of the four focus universities in East Africa [5] were represented by the KIs. Three (3) of the representatives were from medium-valued partners and one (1) from a lower-valued partnership. The non-higher-value representatives were interviewed to gain the international partner perspective on specific unusual partnership issues.

The decision was made to focus primarily on partner universities – 21 of the 26 universities represented in Phase 3 - involved in higher-value partnerships with the four focus East African universities since these were the universities the senior representatives and other representatives of the focus East African universities generally provided the most information about during Phases 1 and 2 of the study, respectively. As noted above and below, representatives from five additional universities were interviewed to gain additional perspectives on issues considered particularly noteworthy because they were unusual.

Approximately one-third (20 of 59) of the representatives were mentioned as a “lead representative” of the partnership by study participants from the four focus universities in Phase 1. Others were identified during the literature review, preliminary discussion or main interview of a lead representative of the partnership from the international university, and during conferences and the work of the lead author (AY). Efforts were made to interview one lead representative of each of the partnerships included but securing these interviews was not always possible due to time constraints and/or logistics. Someone who helped found the partnership from the international partner’s side was interviewed for 63% (19 of 30) of the partnerships included in this sample. Among university administrations involved in the global health work of European and North American universities, only two respondents were interviewed [5].

Fifty-seven (57) of the KIs were current or past representatives of 25 partner universities in nine countries (Canada *n* = 4, Egypt *n* = 1, Germany n = 1, Netherlands *n* = 2, South Africa n = 1, Sweden *n* = 5, Uganda n = 1, United Kingdom n = 1, United States *n* = 9). In addition, two representatives from two universities newly partnered with one of the four focus universities, but not mentioned by their representatives in Phase 1, were opportunistically identified and interviewed.^3^ This was done to gain additional perspective of newer international partners.

### Data collection and analysis

We developed a semi-structured interview guide for the individual in-depth interviews. KIs were typically asked additional questions specific to their partnerships. These questions are not presented in the interview guides to better ensure confidentiality.

Initial interviews were conducted between March 2014 and November 2015. Follow-up interviews were conducted and emails exchanged into 2017 to gather additional details and clarify issues. Interviews were conducted in-person or by phone/Skype by the first author (AY). All interviews were transcribed and analysed.

Rigour was maintained by: conducting fairly lengthy interviews in most cases (interviews lasted between 26 and 145 min, with most lasting between 51 and 78 min); by asking questions on all key issues; and by triangulating with the findings from Phases 1 and 2, peer-reviewed and grey literature and, where possible, other representatives involved in the partnerships from the international partner institution and outside it. In addition, follow-up was done by voice and email communication to clarify anything that was unclear.

Thematic content analysis was conducted [12] on all the transcriptions. One of us (AY) reviewed each transcript and coded them using Atlas.ti 7. The analysis focused on KI responses coded as “Start of Partnership”, “HIC Benefit”, “LMIC Benefit”^4^, “Funding” and “Central Admin Support” for responses from representatives from the 23 European and North American and 3 African partner universities. Themes were then related to Clark’s five elements.

### Ethics, consent and permissions

Ethics approval was obtained for the entire study (Phases 1, 2, and 3) from: the Senate Research Committee of the University of the Western Cape (13/5/15); Institutional Research and Ethics Committee Secretariat of Moi Teaching and Referral Hospital / Moi University School of Medicine; Ethics and Research Committee, Kenyatta National Hospital / University of Nairobi; and, National Institute for Medical Research in Tanzania. Research Clearance was received from the Tanzanian Commission for Science and Technology.

Written informed consent was presented, requested and granted by all participants in this study. The most critical ethical issue was preventing attribution of specific comments to specific individuals since the study included relatively few universities, partnerships and representatives. In this phase of this study, we sought to minimize this risk by increasing the number of partner universities and representatives from them interviewed. In those few circumstances when we felt this standard might not be met, we contacted the individual(s) to determine if they wished to include a clarifying statement or rebuttal. Only when a KI specifically stated that something was “off the record” was it not included. In some cases, the interviewer (AY) specifically asked if a statement was “on record”.^5^

## Results

### Partnership initiation

In some cases, a representative or representatives of the international partner approached representatives of the focus university directly to propose partnering. In other partnerships, a representative of the focus university approached a representative of the international partner. In still other cases, there was an intermediary; for example, a representative of the World Health Organization (WHO), a donor agency, a colleague or a relative who made introductions or encouraged a meeting. Other times, as in the case of Dalhousie University and MUHAS, a director of a nursing programme in an HIC met a former student now based at an LMIC university at a conference and they agreed to address a need through a joint project partnership [13]. Each partnership had its unique history that includes a variety of actors, motivations and serendipitous events. Often the stories are long and rich [14, 15].

Depending on the specific type of partnership [5, 16], the importance of the contextual issues within the East African partner country varied for the international partner. For example, the stability of the country and resulting security for visiting representatives were important in all cases, although the degree of importance varied to some degree depending on whether or not students, especially undergraduate students, were likely to participate in addition to faculty. The ease of obtaining student visas, working visas/permits and/or medical licenses was important depending on the nature of the activities conducted. Some international partners that planned to have their representatives reside on-site for many months or years mentioned that the level of development of the specific locale of the university needed to be sufficient to make it desirable for potential accompanying family members. Other representatives within the same partnerships considered the quality of primary and secondary schooling available if they had children of school age.^6^ A hospitable climate was mentioned by some study participants. Some international partners were interested in a specific area of medicine; for example, ophthalmology, internal medicine, or cardiology.

#### The role of the individual or a small group

An individual or a small group of individuals within a specific discipline are often credited with establishing partnerships. Some partnerships, through persistence, changing context or the value of activities to the portfolio of the international partner, became long-standing or institutionalized at a university and expand to other departments or faculty. Others remained largely on the periphery. Ultimately the challenge was to integrate the work into the core educational and research activities, what Clark calls the academic heartland, of the institution. This is supported by a number of statements by KIs. One North American representative stated:

We knew we'd only get one chance at this [idea of establishing a long-term partnership]. We were not experts in global health. As you know, global health wasn't even a term back then. I think we called it international health or international medicine, these sorts of things. We knew though that we would only get one chance at success here. We were kind of pushing our own school about as far as they could be pushed. Even as far as they could be pushed, even though they weren't really supporting us. These all came from the division. We thought, ‘Let’s go where we think we can be most successful initially, and then try to expand from there.’

A European representative made a similar comment about the support he received from the individual to whom he reported, stating:

I sat down with my head of department and asked, ‘what would you think if we were looking for a partner somewhere in the developing world for long term partnership with the aim of training people there?’ He said, ‘wonderful idea, I'm with you, don't expect too much input from my side in term of letters, work, travel, etc. You do all of that but I support you … .’

### Benefits of partnership

Many international partners were motivated to establish and sustain partnerships with the East African universities by their desire to provide members of their academic heartland, faculty and students, with opportunities to conduct research and to provide trainees with educational opportunities of interest to them. Somewhat less common but still an important theme, however, was the desire expressed by several representatives to be socially responsible. The need to form partnerships to secure grants was also found to be a motivating factor for establishing new international partnerships.

Leadership in the Steering Core was rarely observed to be the initial driver for initiating partnerships but sometimes provided initial support to explore partnerships and/or support to help sustain or further advance them. Oversight in terms of guidance to minimize and manage risks associated with international partnerships activities was also observed by the Central Administration within the Steering Core. The examples below illustrate how these themes were articulated by respondents in recounting the histories and importance of the partnerships to their institutions, units, or programs of work.

*Research* motivated many universities, especially research focused universities, to partner internationally. Representatives, faculty, post-docs and *trainees* (PhD students) from Harvard University all conducted research at MUHAS. Harvard representatives indicated that the university tends to lead with research when it comes to partnering internationally, although training and education activities and public health practice (i.e. knowledge translation) for MUHAS, were also part of the partnership, as was service by way of HIV/AIDS treatment in partnership with MUHAS and the city of Dar es Salaam.

The London School of Hygiene and Tropical Medicine (LSHTM) is upfront when discussing the need for partnerships in LMICs in order to do its work. In its 2014 submission to the Research Excellence Framework, the LSHTM stated, “Partnerships in low- and middle-income countries are also essential for our research aims” before noting that KCMUCo was one of its five principal partnerships globally [17]. This comment was also made by LSHTM representatives in Moshi. Although LSHTM may be principally concerned with achieving research aims through partnerships with universities in LMICs, it was also involved in capacity building activities with KCMUCo such as supporting and training Master’s and PhD students.

Duke University’s partnership with KCMUCo began when, in 1995, a Tanzanian professor at MUHAS in Dar es Salaam moved to Moshi and Kilimanjaro Christian Medical Centre (KCMC) to lead the establishment of KCMUCo. KCMUCo would be the academic arm of KCMC and opened 2 years later, in 1997. The professor asked Duke representatives based at MUHAS if they were interested in partnering with the new medical school. Following this request, a number of Duke representatives started partnering with representatives at KCMC. Duke’s initial focus was largely on experiences for US trainees at KCMC; specifically, providing clinical rotations for US medical residents through the Minority International Research Training Program (MHIRT), although research links were also established at what would be KCMUCo’s teaching hospital.

Some partnerships were driven principally by the desire to be *socially responsible.* This was the case of the partnership between UoN and Ludwig-Maximilian University of Munich (LMU) in Germany, as stated by a representative of another German organisation in the partnership:

The starting point of the initiative of the training relationship between the university and hospitals was basically the relationship between Kenyatta Hospital and University of Nairobi and Munich, and it is down to personal initiative of … [one individual – a German ophthalmologist] who spent time in Africa and started with the idea that it could be a good idea to join the two [universities] together.

The German ophthalmologist had spent 2 years in Mbarara (Uganda) at an upcountry hospital between completing his medical degree and the specialising in ophthalmology at LMU. During his M. Med, he expressed to the head of his department that he wished to return to Africa and “teach so that we can multiply the number of specialists.” With the assistance of the German foreign office, LMU sent letters outlining a proposal idea to German Academic Exchange Service (DAAD) in many countries. Only DAAD representatives in Ethiopia, Kenya and Tanzania replied stating they were interested. The economy of Tanzania was facing challenging times and in Ethiopia, the *Derg* was in power following the overthrow of Haile Selassie I. As a result, Kenya and the UoN were selected. The German ophthalmologist and his family lived in Kenya from 1978 to 1985 to help establish UoN’s M. Med in Ophthalmology. As the partnership matured, trainees from LMU also benefited by means of clinical placements and research.

The desire to be socially responsible by supporting the focus universities in *building their capacity* was also observed at the start of other partnerships, including Dalhousie University (Canada) at MUHAS; Indiana University at MU; University of Toronto at MU; and Radboud University at KCMUCo. In the first case, the partnership implemented a $1.2 M project funded by the Canadian International Development Agency (CIDA) between 1988 and 1993. The Tanzania Nursing Education Program’s principal outputs were nine Tanzanian graduates from Dalhousie University School of Nursing - 6 with bachelors’ degrees and 3 with masters’) - and establishment of a bachelor of science in nursing program at Muhimbili [13].

Representatives of Indiana University desired to focus on building the capacity of a specific type of LMIC institution. One member of the team commented:

Though I could have partnered anywhere, or (at least) in many different places. [Another member of the team] said, ‘No, we need to focus on partnering with another academic health centre.’

Almost 20 years later, the same IU representative would restate his conviction that North American medical schools are best placed to support the improvement of health services in SSA by partnering with academic health science centres (AHSCs). He persuaded a visiting representative of the University of Toronto to Eldoret (Kenya) to return to their university and try to convince its Department of Obstetrics and Gynaecology to partner with MU in Reproductive Health through the AMPATH Consortium, instead of partnering with a district hospital near Lake Victoria.

Other international partner study participants either stated directly or tacitly that it was important to support the development of the focus university and their teaching hospitals as AHSCs, and the tripartite mission of education, research and service that AHSCs embody [18]. A representative of Radboud University in Nijmegen (Netherlands) mentioned how KCMC (the hospital), KCMUCo (the university) and KCRI (the research centre) are now becoming a “university medical center”.

Frequently *more than one motivating factor* was at play simultaneously; for example, trainee interest at a university may drive a university to secure international placements at the same time faculty members want to conduct research and a global health leader is concerned with the whole process being socially responsible. One respondent from a US university expressed this opinion:

[These partnerships] … are really responding to demands first of students. … Overseas engagement … is led one part by researchers but the larger part … [is] student interest. It was really for us a question of how to ethically support an engagement but also how do you ethically provide and ensure that you're just not passing your students off overseas - charging them tuition and making them somebody else's responsibility and relying on their hospitality to do so.

*Donors* increasingly encourage, require and/or support SSA universities to develop the project concepts or have them initiate the partnerships. This is true for both bilateral and consortium partnerships, whether South-South, South-South-North or South-North. A Makerere University representative outlined how USAID used this approach to first bring Makerere and MUHAS together and then other Schools of Public Health in East Africa through Leadership Initiatives for Public Health in East Africa (LIPHEA):

The model by which request for proposals are structured, in such a way that the South to South universities get together to put together a proposal in capacity building that you can then offer to a funder in the Global North is the *creme de la creme* of capacity building. Take the case of LIPHEA, but I had to link up other universities. I brought all deans together. We all have gaps. We sat together to build a proposal. Makerere is strong in Epidemiology. MUHAS is strong in Social Science.

The SSA universities were the leads for The US National Institute of Health (NIH) Medical Education Partnership Initiative (MEPI) [19]. The Fourth Round of the British Council Development Partnerships in Higher Education (DelPHE) required that only higher education institutions in LMICs lead the proposals and “encouraged” South-South and multilateral partnerships. MUHAS prepared the concept note for a recent grant opportunity funded by The Swedish International Development Cooperation Agency (SIDA) [20]. In at least the first two cases, MEPI and DelPHE, some of the successful grants were considered to have been written principally by the Northern partners, albeit in consultation with their SAA partners.

Grantsmanship is, of course, an important issue in the competitive world of seeking, securing and sustaining funding. This was noted by a US study participant who was leading a project that was not focused on HIV or AIDS research but kept making reference to it. The KI stated:

We have to sort of insert HIV periodically into things. [Under a previous project administrator at the organisation it was understood that] ‘… yah, cervical cancer screening. Yes, that's important for HIV. Giving people primary care and screening them for their hypertension and diabetics, that's probably important for HIV infected people.’ Now, everything is put in these buckets … It's really complicated. I see this (the programme I lead) as a global program but I'm also realistic that to get the funding, we have to sometimes direct [our writing] towards an interest [of the donor].

In another example, a Northern university encouraged a South-South partnership. The University of Bergen, using Norwegian government funding (NORAD), contracted the University of the Western Cape (UWC) to help MUHAS develop part of the curriculum for its Globalization and Health course. The initiative for this link came from the Norwegians. MUHAS was supported with a module for its course while UWC benefited from having a module for one of its courses updated.

#### Reverse innovation

While many representatives stated they learned much through their partnerships with the focus universities there was only one, very limited, example of reverse innovation mentioned by a study participant from an international partner. A US professor mentioned that faculty from MUHAS organized a “teaching collaboration” session during one of their visits to the Tanzanian university. The professor stated “It was a really excellent way of getting together with the faculty and exchanging challenges that you were facing in the classrooms and stuff like that.” Faculty from the US university continued with it thereafter in the US. This leads us to the issue of who specifically at the international partner universities is involved, in whole or in part, in establishing the partnership and the perspective that each of these individuals brings based on their values, life experience and the position they hold at their university.

### Findings related to Clark’s four elements

Six primary themes, two of them with two categories each, emerged from our thematic analysis on why partnerships started in terms of desired benefits from the international partners’ perspective. Five of the six themes fit within the first four of Clark’s elements for examining entrepreneurial universities [see Table 1 - Themes for Partnering Organized by Clark’s Elements]. The sixth theme fit with the concept of social responsibility. Illustrative examples of the themes are presented in Table 1 and discussed in the narrative below. At least one of the themes was observed within each partnership, and sometimes all of them were observed within one partnership.

**Table 1:** Themes identified for explaining why universities establish interuniversity global health partnerships organised by Clark’s elements and a new element

Clark Element	Theme Explaining an Interest in Partnering with Focus Universities in East Africa	Types of Activities
***Steering/Managerial Core***, includes central administration, Deans and Chairs	***Internationalisation by way of “global health”***	• Seed funding • Establish policies • Memorandum of Understandings (MOUs) • Prioritize/institutionalize specific partnerships at the department of faculty level. • Visit international partners
***Academic Heartland*** – research & training	Conduct ***research*** • Access to ***expertise (knowledge) or an opportunity*** that their institution or country lacks. • Essential to ***mandate*** ***Education*** – respond to trainee interest	• Towards post-graduate degrees (Master’s & PhDs), publications, expanded research network • Novel research in tropical medicine • Secure sites for ***trainee placements*** (undergraduate and Master’s) for service placements, exposure to research methods, electives, practicums).
***Development Periphery*** – centres and programmes engaged in outreach	***Global Health Centres/Institutes*** explore, develop, coordinate and support activities and partnerships to achieve stated objectives set by the Steering Core	• International partnerships and networking • Provide and support opportunities of interest to Academic Heartland
***Diversified Funding Base*** – additional to traditional government sources and overhead from research grants	***Funding*** ^a^ • Second stream – soft money • Third stream – soft money or discretionary funds	• Grants and contracts from research councils • Local government, philanthropic, foundations, student fees
New Element		
***Global Health as equality*** – belief that quality health care should be available universally.^b^	***Social Responsibility*** • Addressing the higher-burden of disease and health inequity in a manner that builds and/or strengthens health professional programmes in LMICs	• Establishment of new degree programmes • Support the use of new pedagogy institutional-wide • Fully-funded exchange opportunities for students of their international partner • Infrastructure development (help secure funding for new buildings (e.g. hospital, laboratories) • Service delivery (i.e. patient care)

^a^ Federal/national government research grants represent second-stream funding. Clark refers to third-stream funding as, “true financial diversification” [Clark (1998), p. 6)] and states in a later publication “there is no limit to the possibilities of third-stream income in its many substreams” [Clark (2001, p.14]. First-stream funding is government funding from “a governmental ministry” [(Ibid*)* p. 12]

Mainstreaming or institutionalizing the activities of the partnership into the core activities of the partner university’s work, education and research, within a department or formal centre of the university, best ensured that the international partnership will be sustained. Partnerships that commenced with research being conducted by a faculty member were this type of partnership from the beginning. Dartmouth University’s relationship with MUHAS began this way in 2001, “… the partnership started because we were doing clinical research, vaccine trials, looking at TB and HIV co-infection.” Additional research work was conducted, educational placements were made possible and capacity building activities for MUHAS in Hanover, New Hampshire were established.

The importance of having faculty leads was emphasized by a representative of another US university:

I think what matters most in any collaboration … what I've learned over the years, is faculty. Are there faculty with similar interest? Because if there aren't faculty with similar interests, no collaboration will work.

Rooting a partnership in the academic heartland of the university allowed for the possibility that the partnership may be institutionalized at both partner universities. By combining faculty research with trainee experiences, the partnerships grew and were sustained. However, even a partnership that secures significant second- and third-stream funding, offers educational placements for its trainees and publishes numerous papers may not be valued across a research focused university. A representative of an R1^7^ university in the US expressed this reality when stating, “I showed him [my boss] all the stuff, 14 million dollars worth of funding and he says, ‘Great, (but) where is the science?’” While the partnership had secured many grants worth millions of dollars, the results being published weren’t considered important by the individual’s university superior.

“None”, was frequently the initial response to the question, “What support do you get from central administration at your university for the partnership?” Upon reflection, however, many of the study participants admitted they received some support from central administration. Other representatives stated as soon as they were asked that they received support from central administration (a key component of the Steering Core), even if they would have appreciated greater support. This was expressed by a Duke representative involved with KCMUCo:

Even the president of Duke has visited KCMC which was fantastic, the dean of the school of medicine, Bart Haynes the head [of] the Duke human vaccine institute, Mike Merson [Director (2006-2017), Duke Global Health Institute (DGHI)] has been there a couple of times, so I think that in terms of university leadership, we have had quite a bit of support. Is it fully sufficient? No. I would like to have more support.

At Duke University the chancellor provided half the salary for a two-year global health residency while the surgical department paid the other half. At the time of interview, this arrangement was only guaranteed for an additional 2 years^8^ Duke’s partnership with KCMUC was stated to be one of the DGHI’s primary partnerships, as was their partnership with Moi University in Eldoret (Kenya) through the AMPATH Consortium.^9^

There was one notable example of a former member of central administration playing a very direct and larger than usual role in the establishment of a partnership. A former school of medicine dean and chancellor of the University of California, San Francisco (UCSF) was stated to be central to the establishment of the UCSF-MUHAS partnership when he became Executive Director of UCSF Global Health Sciences upon the conclusion of his term as dean.^10^ A number of other UCSF representatives interviewed stated the individual’s participation early on, along with the participation of the vice-chancellor of MUHAS, in the development of the partnership played an important role in identifying a key need for MUHAS and mobilizing representatives across UCSF to implement the project, once a multimillion-dollar grant was secured from the Bill and Melinda Gates Foundation.

In the case of the University of Toronto, a grant from the University’s Academic Initiative Fund provided 2 years of initial funding to the Centre for International Health^11^ to establish the HIV/AIDS Initiative-Africa in 2005. It was the lead of this initiative who first met the Indiana University field director at MU and coordinated approaching the Chair of the Department of Obstetrics and Gynecology to become a partner of MU as a member of the AMPATH Consortium. The department had identified *social responsibility* as an objective in its recently conducted strategic planning exercise and argued that a partnership with MU, focused on capacity building of its Department of Reproductive Health, would help assist in realizing this objective. It was also likely fortunate that the chair of the department was simultaneously the chair of OBGYN at one of the University of Toronto’s teaching hospitals. This facilitated some matching funds in the initial years. Two consecutive 3-year grants from a University of Toronto donor, who had initially encouraged the Director of the HIV/AIDS Initiative-Africa to visit Eldoret, allowed the department to play a leading role in supporting reproductive health at MU.

It is questionable if the development (i.e. fundraising) representatives of Toronto’s faculty of medicine were keen to pursue funding from a key private foundation for international capacity building. It appears likely that when the HIV/AIDS Initiative-Africa was initiated many university administrators desired that the initial support would build research partnerships that would better enable securing grants from pharmaceutical companies and private foundations like the Bill and Melinda Gates Foundation [21]. Ultimately, however, the University of Toronto’s partnership with MU has become one of the faculty’s two featured international outreach activities in “building capacity locally to meet local needs” [22]. Both of these types of global outreach partnerships are in East Africa (the other one is the Toronto Addis Ababa Academic Collaboration or TAAAC).^12^ All the other international partnerships highlighted in the 2014 Faculty of Medicine Annual Report, with Brazil, European Union, China and Australia, focused mainly on research and policy collaborations, except for those in the Middle East that include “contractual agreements”. Moreover, with time, partnerships addressing social responsibility allowed the university to compete for grants - a better fit with research consistent with the academic heartland of universities. In 2013, members of the University of Toronto partnership with MU lead a submission to a Gate’s funded grant that that would have brought together a number of leading researchers from across Toronto Academic Health Science Network.

Dalhousie University, like the University of Toronto, had partnerships in other areas of the world that were based on contractual agreements. These types of partnerships address health inequalities but do not share all the characteristics of collaborative global health partnerships. They are paid consultative or operational partnerships.

As noted a number of times above, partnership representatives looked to research grants and other government agency grants, or second-stream funding as a main source of funding for activities and some *seed funding* was sometimes available from within a university to explore or initiate partnership activities. However, diversified funding, specifically third-stream funding, was identified to be important to initiate new activities before government funding was available, broaden the funding base to support additional activities and sustain current work between government funded grants. Third-stream funding sources were most numerous and of larger amounts for North American universities, especially those in the United States, but there were examples of European universities raising funds from third-stream funding too.

Representatives at Indiana University partnered with Moi University have a long history of securing funding from a variety of sources to broaden and sustain the partnership. For the first eight year of the partnership all activities were self-funded by the Division of General and Internal Medicine in the Department of Medicine of Indiana University School of Medicine in Indianapolis. This was only possible because of discretionary revenue generated by the Division through service contracts with local hospitals, the Department of Corrections and others. All of these funds were deposited in an account controlled by the Chief of the Division. As Division faculty members were salaried all revenue from these service contracts went into the Division’s account. Surplus created discretionary funds for the Division. As the chief of the Division had an entrepreneurial spirt that embraced social responsibility globally and was supported by enough members of the Division, the Division was able to start the partnerships with Moi. In 1998 extramural funding, including private philanthropic support, was secured to support the partnership too. However, the IU representatives again looked internally to secure funding to initiate its anti-viral therapy (ARV) in 2000. ARV therapy for the first HIV patient in Eldoret was initiated after $US10,000 was provided by IU’s infectious disease department in 2000 [[14], p.9].^13^ The initial scaling-up at Moi Teaching and Referral Hospital (MRTH) and Mosoriot Health Centre from 2000 to 2003 was done with a “patchwork” [23] of funding from foundations before the United States’ Presidential Plan for Emergency AIDS Relief (PEPFAR) was launched in 2004. An IU represented stated that “PEPFAR saved us. The whole thing would have come crashing down [without it].” Nevertheless, the diversification of third-stream funding continued after PEPFAR funding was secured with additional support from other private foundations and funders, including pharmaceutical companies.

While one Swedish representative stated there isn’t nearly the same level of foundation funding available in Sweden, a number of examples of third-stream funding for support partnerships were identified. One university secured grants from the Linus Pauling Institute to support reciprocal student exchanges. Another university received money from its County Council through the efforts of an entrepreneurial staff member of the County who was able to bank administration funding for international capacity strengthening partnerships from a nationally funded health program.

#### An additional element: social responsibility

Social responsibility was identified earlier as a reason for the University of Toronto’s Department of OBGYN to explore partnering with Moi University and LMU initiating its partnerships with the University of Nairobi in Ophthalmology. The growing importance of social responsibility to the core mission of another research university was also expressed by the department Chair in a school of medicine at an R1 university in the United States:

Now that we’ve seen the higher quality residents that are attracted to our program, even the faculty who might look a little bit askance at spending money in Kenya understand that it does recruit a different caliber of resident. … when I came [to this university] the residents were ... they wanted to be very well- trained and they wanted to go out and earn a good living. They were American ... typical American physicians, they were not globally minded. ... and now we find … they’re much more interested in local under-served and global under-served [populations], family planning … learning about methods of family planning.

## Discussion

Using Clark’s elements, especially the first four, to examine the establishment and development of global health partnerships, we observed that each of them was important to varying degrees in all of the partnerships explored here. It is worth underlining that Clark’s model sought to categorize an initially limited number of universities which were different from traditional universities. In our study, all of the universities would be considered “entrepreneurial” and the elements that were emergent less than 20 years ago – such as third-stream funding – are now completely mainstreamed. Representatives of research focused universities may be more likely to be solely focused on research towards scientific development, especially biomedical research, and research to support PhD candidates, but our findings show the interaction between the various elements of a university – the Steering Core, the Academic Heartland, Development Periphery, Diversified Funding – to start, build and sustain partnerships is often a process of back and forth. Nevertheless, these interactions and the imperatives of funding and internationalization appeared always to be manifested through or framed in terms of the most traditional roles of the university: teaching and research. Clark’s “academic heartland” was never the sole player in any partnership, but partnerships that did not resonate strongly with the academic heartland did not endure.

There is little doubt that sustaining a specific partnership depends on the energy of at least one core individual, although to grow and sustain it a small group of committed representatives is required and future leaders must be developed too. The future leaders can come from various backgrounds, but most of international partners of the higher-value partnerships of the four focus universities in our study included representatives who had on-the-ground experience in an LMIC as trainees at some point.

Individuals often play an important role in interuniversity global health partnerships. Social responsibility under the rubric of global health is but one of many areas of importance for many universities today. Interested chairs of departments can use their authority and discretionary funding to support the establishment of partnerships, guide members of the academic heartland (especially faculty) into a partnership, and provide continued leadership. Chairs, even ones who aren’t directly involved in a partnership, can support a partnership by committing, for example, to assist junior faculty members by committing a post in the department upon completion of a long-term overseas placement. While this may be more difficult today than it was in 1980s West Germany, the general idea remains valid. Finally, individuals with previous international experience, but who may not be researchers, were found to have the coordination skills often needed to bring academics together to achieve common goals internationally. As Pinto et al. [24] note, “Such coordination is rarely supported centrally by the institution and may take academics away from their primary activities with partners.”

Social responsibility appears to be important at some of the international partner universities, or at least among a pool of representatives within some universities, and is being mainstreamed into their objectives. Social responsibility within global health may be the equivalent of being *entrepreneurial* in Clark’s work. Social responsibility speaks to the “global health partnership movement” [25] or global health “solidarity” [26] envisioned by some.

It is important to remember, however, that most of the international partnerships included in this paper were perceived to be of higher-value by the focus university representatives [5]. In addition, perspectives sometimes varied significantly between individuals within the same university. For some individuals, social responsibility was not a concern or remained secondary to the core objectives of educating the universities’ own students and supporting their own faculty in discovering new knowledge through research. The leaders of only some of the partnerships at the international universities may embrace the norms of collaborative partnerships at the international partner universities. Others may continue to either parachute researchers [27], remain part of an isolated unit on the development periphery or be isolated within departments or faculties for a variety of reasons including time or financial constraints, lack of leadership from the steering core, or individuals having different values.

Duke University created the Duke Global Health Institute (DGHI) in 2006 and it may be a good example for how to bring units of a university together under one roof to help to institutionalize global health across a campus. Its approach can be compared to other universities with perceived higher-value partnerships in this study that also expanded their Developmental Periphery with global health centres during the first fifteen years of this century - namely UCSF [28] and University of Toronto [29, 30], other North American universities that have established similar centres [31, 32]; and other universities approach global health in other regions of the world, including LMICs [33].

Although there is an increasing body of literature within global health about the potential for “reverse innovation” [34, 35] or “frugal innovation” [36] within North-South partnerships improving health practices or systems in HICs, this study did not identify any illuminating examples. Further, reverse innovation was not identified as an element or theme for the international universities to partner with the focus universities. The benefits to be realized through the partnerships were seen to be largely and directly for the individuals and institutions involved themselves.

### Limitations and further research and analysis

The most significant limitations of this study are that only one or two representatives were interviewed at 15 of the 26 institutions and that only two representatives not directly involved in the partnerships participated. However, the “perspective” of non-direct representatives was offered by some study participants in their answers and obtained from official documents of the universities. Further research on the specific sources and amounts of funding – actual and in-kind – is warranted to quantify the levels of diversified funding. While third-stream funding is likely to be able to sustain partnerships activities to some degree it is unlikely to enable the level of research, education or service activities envisioned by many for ‘significant’ partnerships.

Another potential limitation was not examining university-affiliated non-governmental organisations involved directly in global health, particularly in the US. For example, Jhpiego, is “an international, non-profit health organization affiliated with The Johns Hopkins University” [37]. These appear to be permanent organisations so would not be considered part of a university’s development periphery, but a representative of one of the SSA international partner universities noted that they secure much US government funding. It was observed that Jhpiego has an office in Dar es Salaam.

Finally, this paper focuses on the perspectives of the international partners. An earlier paper examined what the East African partners valued [5]. Research is now required on the extent to which international, interuniversity global health partnerships can address the respective needs of all parties involved while striving to improve health and achieve equity in health for all [38], as many universities accept [39–41].

## Conclusion

A wide variety of universities are involved in global health partnerships. Universities worldwide enter partnerships for a variety of reasons, notably for research and training benefits to their own staff and students, for social responsibility reasons, and to respond to funding opportunities and imperatives. It is important to examine the specific interests and values of the individuals involved and where they are based within their university’s structure to more fully understand their motivations. Burton Clark’s framework of “entrepreneurial” universities offers a useful, robust approach to analysing the diverse and sometimes divergent interests and motivations for international partnerships in universities facing the imperatives, constraints and opportunities of globalization. Conduct of more empirical research on the processes used to establish and sustain global health centres at many universities would be timely.

## Footnotes

1 Clark refers to this as “an integrated entrepreneurial culture” [(Clark, 1998), p. 5]. The key issue for an entrepreneurial university is that change from traditional ways of doings things and structuring a university is embraced (Ibid*,* p. 7).

2 Specifically, “lower middle income” or “upper middle income”.

3 One of these two KIs was from one of the partner universities identified by the KIs interviewed in Phase 1 of the study. The other KI was from a university not identified by any KI in Phase 1. Therefore, KIs in Phase 3 were from a total of 25 of the 125 partner universities in Phase 1. However, some of the KIs interviewed in Phase 3 were from the same universities but involved in partnerships at different focus universities.

4 In the coding, “LMIC” was used for specific benefits for perceived benefits for the international partners from Egypt, South Africa and Uganda. “LIC” was the code reserved for perceived benefits for the focus universities.

5 Competing interests: The lead author (AY) was employed by the University of Toronto as its Program Manager - AMPATH-UofT when the data for Phases 1 and 2 of this study were collected.

6 Locations with primary and secondary schools with International Baccalaureate® (IB) programmes – see http://ibo.org/ (accessed 30 October 2017) would likely be better able to attract some long-term placements

7 R1 refers to the top level of research universities in the Carnegie Classification of Institutions of Higher Education in the United States. These are doctorate-granting universities. See-https://en.wikipedia.org/wiki/Carnegie_Classification_of_Institutions_of_Higher_Education (accessed 26 October 2017).

8 To date, Medicine, Emergency Medicine, Psychiatry, Radiation Oncology, Pediatrics and Surgery residents have participated.

9 Both the KCMUCo and Moi continue to be primary partners of Duke. There are now 12 “priority partnership locations” listed on the Institute’s web-site, https://globalhealth.duke.edu (Accessed, 29 Oct 2018).

10 See: http://history.library.ucsf.edu/debas.html. (Accessed 26 October 2017).

11 The Centre for International Health, or CIH, was an informal centre within the University of Toronto making it characteristic of the type of units typical of the development periphery at universities: easily established and easily de-established. The CIH was dissolved in 2009. After it was dissolved the Office of Global Health was established within the Dalla Lana School of Public Health. In 2012 the Institute for Global Health Equity and Innovation (IGHEI), a university-wide entity, was founded. The IGHEI is housed within the DLSPH.

12 See: http://taaac.com/. The founder of TAAAC was a VSO volunteer in Ethiopia in the 1970s

13 One senior representative of the IU/Moi partnership recalls that the infectious disease department’s total support of the ARV project was US$60,000.
